# The University of Buffalo

**Published:** 1880-03

**Authors:** 


### EDITORIAL.

---

### THE UNIVERSITY OF BUFFALO.

### THE MEDICAL COMMENCEMENT.

THE winter season of the Buffalo Medical College closed with unusually interesting exercises, which was made the occasion to invest a class of fifty-six graduates with the degree of Doctor of Medicine. Not only here, but elsewhere, the season has yielded an abundant harvest of candidates for the labors and the honors of the Medical profession.

The fact is significant, however, in the experience of the present session, that the higher standard of requirements for graduation and the increased facilities, with the lengthened term of lectures, afforded for acquiring a theoretical and practical knowledge of the science of medicine, have met an emphatic endorsement by the profession.

We desire, especially, to direct attention to this important result. The leading medical schools are extending their lectures, and establishing a graded curriculum of study with regular and frequent examinations. The discussions of late upon the subject of medical education, and the favorable action of medical societies have imparted an unusual impetus to this important movement. The authorities of the Buffalo Medical College, composed of our most progressive men, we think acquiese heartily in this improved sentiment. The result, as unfolded during the last session, justifies the profession in extending its approval and support to this and other institutions, whose aim it is to meet the demand for a higher standard of medical education, and thus, to prove faithful custodians of the trust committed to them in granting medical degrees.

The agitation and discussion of this question places the future status of the medical profession, where it justly belongs, (1) upon the members of the profession in their choice of students, and (2) upon medical colleges in affording ample facilities for clinical and didactic instruction, and in exacting a rigid examination on the basis of the higher standard of requirements, which it is their purpose to establish.

Such evidences of liberal and progressive ideas and efforts prevailing throughout the country in behalf of the most important interests of the profession, plainly demonstrate that the present decade will do much in the solution of this hitherto intricate problem. We are not less ambitious than the authorities to which the Buffalo Medical College is intrusted, that they should be, in the future as in the past, in the vanguard of medical reform. Every movement in advance is met by a generous response and support. The late commencement was a most flattering exhibition of its power and influence. The alumni, old and new, are highly creditable representatives of the profession and of their Alma Mater.

The position of the JOURNAL upon this and kindred questions, affecting the present and future of the medical profession, will be that of earnest advocacy of every step in advance, and of approval of whatever concerns such vital interests as that of medical education.

